# Case Report: Dostarlimab for treatment of aggressive cutaneous squamous cell carcinoma

**DOI:** 10.3389/fmed.2024.1322210

**Published:** 2024-03-11

**Authors:** Sophia Gandarillas, Horace Tang, Bahar Dasgeb

**Affiliations:** ^1^Department of Dermatology, Wayne State University, Detroit, MI, United States; ^2^Department of Hematology, Community Medical Center, Toms River, NJ, United States; ^3^Department of Surgical Oncology, Rutgers Cancer Institute of New Jersey, New Brunswick, NJ, United States

**Keywords:** cutaneous squamous cell carcinoma (cSCC), dostarlimab, treatment, cemiplimab, standard of care, KMT2D, keratinocyte carcinoma, recurrent squamous cell carcinoma head and neck

## Abstract

Cutaneous squamous cell carcinoma (cSCC) is the second most common malignancy with the aggressive cSCC subtype being especially worrisome due to its higher metastatic and mortality rate. An 80-year-old immunocompetent Caucasian man presented with a locally advanced and recurrent cSCC for which he underwent six Mohs surgeries, radiation therapy, and standard immunotherapy treatments. Throughout treatment, the patient’s cancer continued to progress across different regions of the face. Biopsy and analysis were performed and showed that the cSCCs had a high mutational burden and oncogenes known to be present in tumors with aggressive nature. After the algorithmically applied standard of care failed to cure or control the progressing disease, the genetic analysis favored dostarlimab as a suitable option. With only three doses of 500 mg dostarlimab q3 weeks, the patient showed a fast response with macroscopic resolution of clinically discernible disease of, the previously noted, locally advanced cSCC on his right forehead, as well as other primary keratinocyte carcinomas on his left contralateral face, nose, left leg, and neck. This remarkable case can present an option for complex patients with locally advanced and recurrent cSCC who failed the current standard of care. Moreover, it warrants a proper clinical trial to assess efficacy and potential indication of dostarlimab in such patients. Of note is the presence of a KMT2D mutation and its well-identified correlation with mismatch repair deficiency (dMMR) and poor prognosis, which can play an informative role in clinical decision making and precision therapeutic choice at the point of care.

## Case presentation

An 80-year-old immunocompetent Caucasian man presented with a locally advanced cutaneous squamous cell carcinoma of the left forehead extending into the left frontal scalp. He was initially treated with six Mohs surgeries which all led to local recurrence. After the last Mohs surgery, he underwent radiation therapy, which also failed, followed by local recurrence within a year. Initial staging with PET/CT scan did not show head and

neck lymphadenopathy or distant metastasis. However, additional primary keratinocyte carcinomas, which had developed in the interim, were detected on PET/CT as discernible FDG avid foci on his nose, ipsilateral (left) cheek, as well as left leg. Histopathology showed that except the tumor on his nose, which was BCC, the remainder of the lesions on his left forehead were primary cSCCs. Therefore, he was started on systemic immunotherapy to treat the above clinical presentation with locally advanced numerous tumors as detailed below.

## Results

**FIGURE 1:**
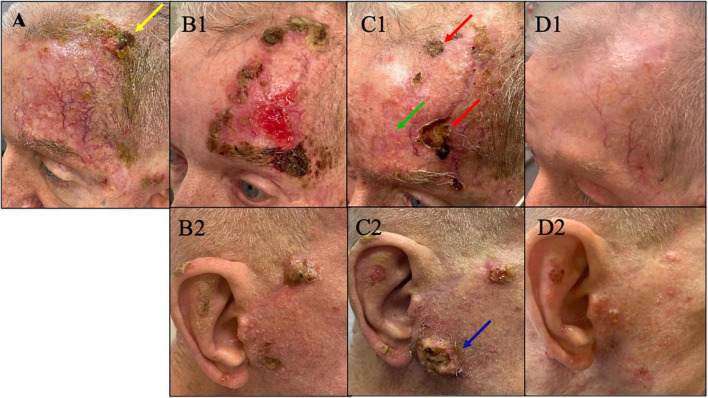
**(A)** Plateau of response with presence of residual cSCC (yellow arrow) on his left forehead after 3 months on cemiplimab. At this point treatment was switched to cetuximab. **(B1,B2)** On cetuximab, the disease progressed on his left forehead and new tumors developed on the contralateral (right) face/ear. At this point pembrolizumab – immunotherapy – was added to his current cetuximab to proceed with combination therapy. **(C1,C2)** After 5 months, despite the initial improvement of the tumor on his left forehead with combination therapy showing a favorable decrease in tumor volume on his left forehead (green arrow), the response plateaued with persisting non-resolving tumors on his left forehand seen in panel **C1** (red arrow). Meanwhile, the disease on contralateral (right) face/ear continued to progress on combination therapy of cetuximab and pembrolizumab (blue arrow). At this point he was switched to dostarlimab. **(D1,D2)** Remarkable response with no discernible clinical cSCC on left forehead and minimally discernible residual disease on contralateral face/ear after just three infusions of dostarlimab. Today after 1 year, this patient continues to tolerate dostarlimab infusion with no limiting adverse events with no clinically detectable disease.

Accordingly, cemiplimab was started as the first-line of systemic treatment to cover the locally advanced cSCCs as well as the BCC on his nose. He initially showed favorable response to cemiplimab and the tumor volume on his left forehead decreased. However, after 3 months, he showed plateaued response to cemiplimab monotherapy with persisting residual disease on his left forehead (yellow arrow in Figure 1A). Therefore, he was switched to cetuximab monotherapy. After 3 months, not only did he show continued progression of the disease on his left forehead, but he also developed new primary cSCCs on his contralateral (right) face/ear (Figures 1B1, B2).

**FIGURE 2:**
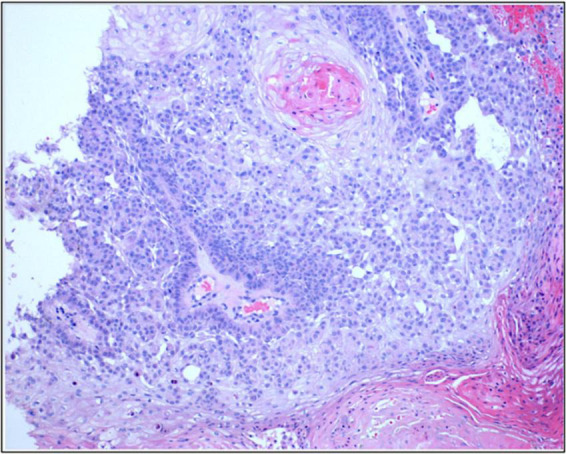
Photomicrograph of the presented patients form the preauricular lesions seen in Figure 1C2 (blue arrow) to confirm that the progressing skin lesion while on combination of cetuximab and pembrolizumab is indeed cutaneous SCC.

Consequently, he was started on combination therapy with continued cetuximab and addition of immunotherapy – pembrolizumab based on the patient’s high mutation rate of 39 muts/mb. Despite an initial favorable response in the first 5 months (green arrow in Figure 1C1), the response plateaued on combination therapy with persisting non resolving tumors on his left forehead (red arrows in Figure 1C1). Moreover, the disease on his contralateral (right) face/ear progressed with new tumors. One of the new primary tumors on his right pre-auricular region (blue arrow in Figure 1C2) was biopsied to confirmed that the progressing disease at multiple sites remains to be cSCC as depicted in the photomicrograph of the histopathology of the biopsied lesion seen in Figure 2. With histologic confirmation of the progressing disease while on combined cetuximab and pembrolizumab, this combination therapy was discontinued. Genetic analysis was done by Foundation One on the above-mentioned biopsied tumor (Figure 2). According to the Foundation One report, dostarlimab was started (Table 1). To our astonishment, all locally advanced cSCC on his left forehead and right face/ear as well as one solitary biopsy proven BCC his nose, all showed a remarkably fast favorable response with no clinically discernible disease on his forehead and minimal residual tumors on his right face/ear with no interim development of new tumors after three infusions of dostarlimab 500 mg q3 weeks (Figures 1D1, D2). Today, after 1 year, this patient continues to tolerate dostarlimab infusion with no evidence of disease progression.

**TABLE 1:** Genetic profile of the patient’s aggressive cutaneous squamous cell carcinoma with a high mutation burden of 39 muts/mb.

	Gene						
	BRCA2	CDKN2A/B	KMT2D (MLL2)	fNOTCH1 splice site	NOTCH2 splice site	TERT promotor	TP53 splice site
Mutation	F1870fs*4	p16INK4a E61* p14ARF G75V	R2922Q	1100-1G>A	31383+1G>A	-139_-138CC>TT	375+1G>A P71fs*52

## Discussion

Cutaneous squamous cell carcinoma (cSCC) is the second most common malignancy in humans, after basal cell carcinoma, presenting with an annual incidence of 1.8 million in the US (1, 2). The reported metastatic and mortality rate of cSCC varies at 2%–5% and 1%–2%, respectively (1–3). Aggressive cSCC is a worrisome subtype with higher metastatic and mortality rate affecting the elderly men, immunosuppressed, and patients with underlying genetic diseases such as epidermolysis bullosa compared to general population. Additionally, aggressive SCCs follow a distinct disease course, and distinct histologic and clinical characteristics. Such characteristics include poor differentiation, desmoplastic features, vascular and neural invasion, and depth of >6 mm represent aggressive nature of cSCC. Clinically, aggressive cSCC tumors are ulcerated, >2 cm, and often occur on the sun damaged skin of the head and neck of elderly Caucasian men. These tumors have a higher risk for metastasis, unfavorable response to standard treatments, and eventually end in mortality (2, 3).

It has been shown that aggressive cSCC are driven by mutational variants that are distinct from non-aggressive cSCC. In addition to displaying a high mutation rate, which would favor immunotherapy as the primary treatment, aggressive cSCC been reported to be associated with many tumor suppressor gene mutations. Of those tumor suppressors, TP53, BRCA2, CDKN2A, NOTCH1, HRAS, CASP8, and NOTCH are well reported and overlap with head and neck mucosal SCC (mSCC). Additionally, more novel tumor suppressors genes, including KMT2C, were also frequently found in aggressive cSCC. While TP53 and BRCA2 are the most frequently found tumor suppressor gene mutations in aggressive cSCC, KMT2C is often associated with invasiveness and poor outcome. Of note, TP53 is detected in almost 100% of aggressive cSCCs based on multiple reports. The aforementioned spectrum of oncogenes seen in aggressive cSCC shows striking similarity to head and neck mucosal SCC favoring immunotherapy as the first line treatment of choice. Moreover, a precise understanding of individual patients’ driving mutations is prudent to execute an effective targeted treatment should immunotherapy fail in complex patients, which are characterized by recurrent, numerous, and aggressive disease (4, 5).

Of key interest is KMT2D, a tumor suppressing methyltransferase. KMT2D has been found to be mutated in 62% of all cutaneous squamous cell carcinomas and associated with more aggressive and highly metastatic phenotypes (5–7). KMT2D was found to be a key driving mutation in endometrial cancer which responds well to dostarlimab (8, 9). Dostarlimab is an anti-PD-1 monoclonal antibody, which was first recognized for its remarkable favorable response in all 12 of the study’s patients with locally advanced colorectal carcinoma with DNA mismatch repair deficiencies (10). Thereafter, dostarlimab has shown favorable outcome as an effective treatment for a wide range of carcinomas such as endometrial, breast, ovarian, adrenocortical, and mucosal head-neck carcinoma, as well as melanoma (10, 11). Once again, studies indicate a meaningful correlation between KMT2D mutations and immune check point-related or mismatch repair genes (12). The well-reported efficacy of dostarlimab in treatment of malignancies with mismatch repair deficiency (dMMR) led to its FDA approval for advanced solid tumors with dMMR (13). Given the identified correlation between KMT2D and mismatch repair genes, it is prudent to investigate gene mutations in advanced and recurrent keratinocyte carcinomas. KMT2D is not only a reliable prognosticator but can also be an informative biomarker for therapeutic decision making at the point of care.

**FIGURE 3:**
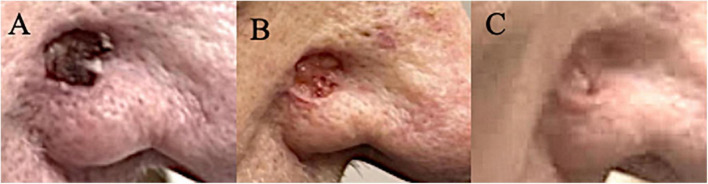
**(A)** One BCC on right nose which continued to grow on cemiplimab and combination of cetuximab/pembrolizumab. **(B)** Noticeable improvement after the first dostarlimab infusion. **(C)** No macroscopic disease that is clinically discernible after two infusions of dostarlimab.

The presented patient had undergone an algorithmically applied standard of care including Mohs surgery, radiation, and targeted systemic therapy with cetuximab combined with immunotherapy. It was not until dostarlimab was begun that the patient’s multiple keratinocyte tumors began to respond. It is worth mentioning that the only non-squamous carcinoma, BCC, on his right nose continued to progress on cemiplimab and pembrolizumab until it responded to dostarlimab (Figure 3). To the authors knowledge, there is evidence in the literature to suggest favorable response of cutaneous keratinocytes carcinomas, SCC and BCC, especially those resistant to the standard of care. This remarkable case warrants a proper investigation and clinical trial to assess efficacy of dostarlimab in treatment of complex keratinocyte carcinomas defined by their locally advanced, numerous, and recurrent nature.

## Data availability statement

The original contributions presented in this study are included in this article/supplementary material, further inquiries can be directed to the corresponding author.

## Ethics statement

Written informed consent was obtained from the individual(s) for the publication of any potentially identifiable images or data included in this article.

## Author contributions

SG: Writing – original draft, Writing – review & editing. HT: Data curation, Writing – review & editing. BD: Conceptualization, Data curation, Formal Analysis, Investigation, Methodology, Project administration, Supervision, Writing – original draft, Writing – review & editing.
